# Relationship between spatial pattern and function of urban land use in Changchun, China

**DOI:** 10.1371/journal.pone.0291121

**Published:** 2023-09-08

**Authors:** Qingxi Shen, Xue Tan, Xipeng Li

**Affiliations:** School of Economics, Jilin University of Finance and Economics, Changchun, China; Wuhan University, CHINA

## Abstract

The urban spatial structure in this study refers to the combination of different categories of land use, and the purpose of the study is to reveal the intrinsic correlation characteristics between urban land use structural combination forms and urban functions. Through the integration of land and population maps and other multi-source data, with the help of exploratory spatial data analysis and other models, this research deals with the land use spatial structure characteristics of Changchun city and its coordination relationship with urban functions. Main conclusions of the study are as follows. The overall density of the land use in the central urban area of Changchun shows patterns of the core being higher than the periphery, the large-scale agglomeration being significant and the small-scale relatively scattered, and the pattern of the mixed land use function index has obvious differentiation characteristics. The study shows that, in the context of the spatial pattern, the overall coupling coordination degree of the land use structure index and the urban function index shows a trend of a gradual decrease, from the core to the periphery. In the context of category differences, the coupling coordination of the land use structure with the population distribution and the Baidu thermal distribution is relatively high, and the coupling coordination with various service facilities is relatively low. Finally, in the context of scale differences, all types of coupling coordination degrees have significant sensitivity to the spatial scales. A large scale significantly reflects the overall decrease in the coupling coordination degrees from the core to the periphery, while a small scale shows the polycentric pattern characteristics of the urban spatial structure.

## Introduction

High-quality socio-economic development has recently become a direction that is actively advocated by the Chinese government in the new era. Consequently, cities as the concentrated areas of the socio-economic development, are also facing the fundamental task of improving the quality and increasing the efficiency of this development. At present, the imbalance of function matching, traffic congestion, and ecological environment destruction in China’s big cities are prominent problems [1–3], as well as crucial causes of the low quality of the spatial structure development in those cities. The spatial structure of the urban land is the projection of the urban functional areas and their territorial connections [4], together with its degree of functional mixing, which is closely related to various urban problems [5–8]. This is in turn directly related to the spatial distribution of human activities and, therefore, the optimization of the urban land structure becomes an important basis for improving the quality of the urban functional development.

Research on the spatial layout and the interaction of the urban functions emerged in the 1920s and 1940s, with the urban zoning ideas of Corbusier, the Athens Charter, the “organic evacuation theory” of Saarinen, and the “concentric circles, sectors, and multiple nuclei" of the Chicago School of Ecology. The three major theories of the Chicago School of Ecology are the most prominent achievements in the study of urban functional space. After World War II, especially since the 1960s, in the face of urban overspill and overcrowding, what has become a central question of the research and planning is how to decentralize the over-concentrated industries and the population in large cities has become a real problem.

The satellite city construction originated from Howard’s idea of the “idyllic city” and the three generations of the new city planning represented by the British have strongly promoted the theoretical practice of the urban functions’ spatial adjustment. At the same time, in response to the inadequacy of the Athens Charter, which overemphasized urban zoning, the Machu Picchu Charter focused more on the organic organization of urban functional space and the creation of an integrated and multifunctional urban living environment.

“The Death and Life of Great American Cities” (1961), written by Jane Jacobs, a renowned urban scholar and commentator, depicts vibrant communities in which multiple urban functions converge, and, as such, challenges the traditional idea of the strict urban zoning. Since the 1990s, under the guidance of the functional zoning, many major cities in China have planned and built numerous large and single-function residential and industrial areas, which have seriously fragmented the connection between the urban functions. This has not only caused an inconvenience for residents, but it has also increased their reliance on motorized transportation and limited the urban residents’ quality of life [6,8].

By taking into consideration the problems arising from the strict urban functional zoning, academics have turned more of their attention to the issue of the urban functions’ spatial integration in recent years [6]. Moreover, the use of mixed land in the countries with high urbanization levels has even been considered as a significant contribution to solving “urban diseases”, such as traffic congestion [8]. Furthermore, when it comes to the regulation of the spatial structure of urban land use two undoubtedly key points of discussion in academic circles have been the questions of how to determine the degree of the mixed urban land use structure and what kind of intrinsic correlation it has with the urban functions.

The exploration of the spatial correlation characteristics between the structure of the urban land’s use and its functions, followed by the provision of guidance for the quality improvement of the urban function and space development has become a theoretical topic and a key element of urban construction. Through extensive research of the existing literature, we have found that the academic research on the urban functional space-related issues mainly focuses on the following four aspects.

The first one is the research on the spatial structure and pattern characteristics of the urban land use. The spatial pattern and the evolution of the urban functions have always been the main topics of urban geography and planning. With the wide application of the remote sensing data and the rise of the spatial measurement methods, research topics such as the urban land expansion and sprawl [4,9–12], urban land structure and pattern evolution [13,14], spatial patterns of the industrial or residential land and its structural changes [15–19] have received continuous attention. It is also important to take note of the recent extensive research on the spatial structure of the urban land and commuting efficiency [20,21], population dispersal [22,23], social equity [24,25], facility distribution [26], and even the impact of the carbon emissions [27,28]. This research has expanded the traditional in the aspect of the spatial structure and pattern characteristics of the urban land. Some scholars have also attempted to study the urban spatial structure of the United States through social survey employment data. For example, Shlomo Angel et al. found that most jobs in the United States are no longer the traditional urban core types [29], which could be a reflection of the new urban spatial structure characteristics present in the United States.

The second aspect is based on the research of the spatial integration characteristics of the urban land functions. The concept of the mixed-use development of the urban functions was proposed as a response to the functional division found in the Western urban design and planning of the 20th century. Under the guidance of the strict functional zoning ideas, many large cities in Europe and the United States have built many functionally divided urban areas, which has led to serious urban problems such as the urban congestion, major pollution, and loss of vitality. This chain of events has then triggered an extensive exploration within the academic community [30,31].

In recent years, active guidance for the integration and diversification of the urban functional spaces, along with the promotion of their integrated development and the agglomeration of population, industry, and other factors has been identified as an academic consensus, with the goal of achieving vitality enhancement and sustainable prosperity of cities [7,8,32,33]. The relevant research has been explored for mainly two reasons–one is to find the research already done on the structure of the urban land, done by applying mixing measurement methods, and through the derivation and comparison of various methods, all with the goal to explore the theoretical and the empirical urban land use structure mixing measurement methods [32,34], and the other is to delve into the interaction or the mixing pattern characteristics between the urban land use functions[30], especially within the “production-living-ecological” spatial interaction, and the pattern characteristics, in order to determine which are more common [35,36].

With the growing imbalance of the urban functional space in China in recent years, the issue of the coordination of the urban functional structure has got more attention in academic circles, especially when from the work-life balance perspective [2,29,37], and thus can be regarded as an important supplement to the study of the integration characteristics of the urban functional space. Unfortunately, the existing empirical studies mainly focus on the interrelationship of the urban space and certain types of spaces, such as the industrial space, residential space, service space, ecological space, etc. [24,29,33,36]. What is more, systematic studies on the interactions of the major functional spaces within cities are still rarely done.

The third focused aspect of the research on the urban functional space-related issues is the benefits of the spatial structure of the urban land use association. The study of the socio-economic effects of the evolution of the spatial structure of the urban land represents a significant starting point for the interpretation of the rationality of the cities’ spatial structure. Relevant studies mainly focus on three aspects. First, the impact of the urban land spatial structure on the overall development or the quality of a certain aspect of the city is discussed through the construction of an index system that reflects the quality of the urban development [1,38]. For example, Zhe Cheng et al. analyzed the main characteristics of the mixed suburban land development in China in terms of its nature, function, and development pattern, and concluded that the mixed use of the urban land can help promote a high-quality development of urban functions [6]. Second, the relationship between the urban spatial structure and the transportation efficiency [20,21], labor productivity [39], industrial land efficiency [40], municipal expenditure efficiency [41], and the economic development efficiency [42] has been analyzed in an attempt to uncover an efficient urban spatial structure. Third, the relationship between the overall urban spatial structure and the economic development performance was studied as well. What has been found is that, while different urban spatial structures were associated with different performance [43], most scholars have still stated that the dependence of the urban economy on the spatial structure and the mixed urban spatial structure of the polycentric cities has, in fact, helped to improve the level of the urban economic performance [44,45]. However, others have claimed that a single center possesses higher economic efficiency through measurement [39].

The fourth and the final aspect is the one of the impacts of the urban spatial structure on urban dynamics. Due to the concerns about the decline of inner cities in the traditionally developed countries and the vacancy of new areas in the emerging developing countries, the issue of urban vitality has recently attracted extensive attention. Most of the relevant studies have dealt with the relationship between the urban land use characteristics and the urban vitality in the discussion of whether the spatial structure of the urban land is reasonable [31,33,46–48]. For example, Jiawen Yang analyzed the non-linear relationship between the land use and urban vitality [31], and Jessica Ferm argued that the mixed land use found in the process of the industrial land reuse can effectively enhance urban commercial development and urban vitality [33]. Moreover, Xin Guo has, based on the street-scale multi-source big data mining and utilization, come to the conclusion that the improvement of the diversity and balance of the urban land use structure will help the improvement of the stability of urban vitality as well [46]. Overall, most of the experts believe that a mixed use and a diverse structure of the urban land use could help to improve the urban vitality.

There is an abundance of research done on the spatio-temporal pattern characteristics of the urban land use, the integration characteristics, the association effects, and the effects on the urban vitality where more evidence supports the necessity of the spatial integration development of the urban land use. The existing studies have focused on the application of remote sensing images, POI data, electronic, and thermal maps, and they are all based on the quantitative spatial analyses [9,19,31,37,46,47], which stem from a wide range of methodological and theoretical references, given in this paper as well.

However, most of the existing studies have analyzed the spatial or the functional spatial characteristics of the urban land use separately, and the relative unity of the data cannot reflect the functional characteristics of the city comprehensively. Thus, the research objects have mostly been discussed at an overall or a regional scale of the city, and, in parallel, research on the spatial scale effects within the city is still relatively scarce, with only few studies providing us with a systematic analysis of the quantitative relationship between the urban land use structure and the urban functions. Furthermore, there are many blind spots within the existing theoretical understanding of the practical guidance for the urban functional spatial reconstruction, which is additionally not favorable to the coordinated evolution of urban land use and functions. Finally, the proposition of a path for the spatial reconstruction of the urban functions, solely based on the interaction mechanism of the “structure-function”, has arisen as an urgent theoretical issue for further exploration.

In consequence of the previous urgency, by taking Changchun City, Jilin Province, China as an example, our study aims to employ multi-source data in creating a database that would reflect the structure and the function of the urban land use. On the basis of the multi-scale analysis of the land use patterns characteristics found in the major functional spaces in the cities, we proceed to study the correlation characteristics of the land use structures and the functional spaces in large cities, and analyze the correlation law of the spatial “structure-function” in large cities, to then, finally, explore the management solutions for the imbalance of the large cities’ functional space matching and the management strategies for the functional space quality improvement.

There has been research on the spatial patterns and functions of land use of big cities, but the innovation brought in this paper is mainly reflected in the multi-scale comprehensive quantitative analysis of the coupling coordination relationship between the urban land use structure and the function. The significance of the study is in the construction of an index system for studying the coupling relationship between the urban land use structure and urban functions, and spatially visualizing the results of the quantitative analysis of the coupling relationship between the urban land use structure and urban functions, which provides strong evidence for the scientific understanding of the “structure-function” coupling relationship of the big cities and provides a new perspective for the evaluation and reconstruction path of functional space quality of big cities.

## Materials and methods

### Study area

A central urban area consists of the core area and the peripheral suburban area, which cover the main urban functional areas from the geographical perspective. The central urban area has been selected as the object of our research as our study aims to provide a comprehensive reflection on the coupling relationships between the spatial pattern and the function of land use in the core area, as well as the peripheral expansion area of a typical city.

**Fig 1:**
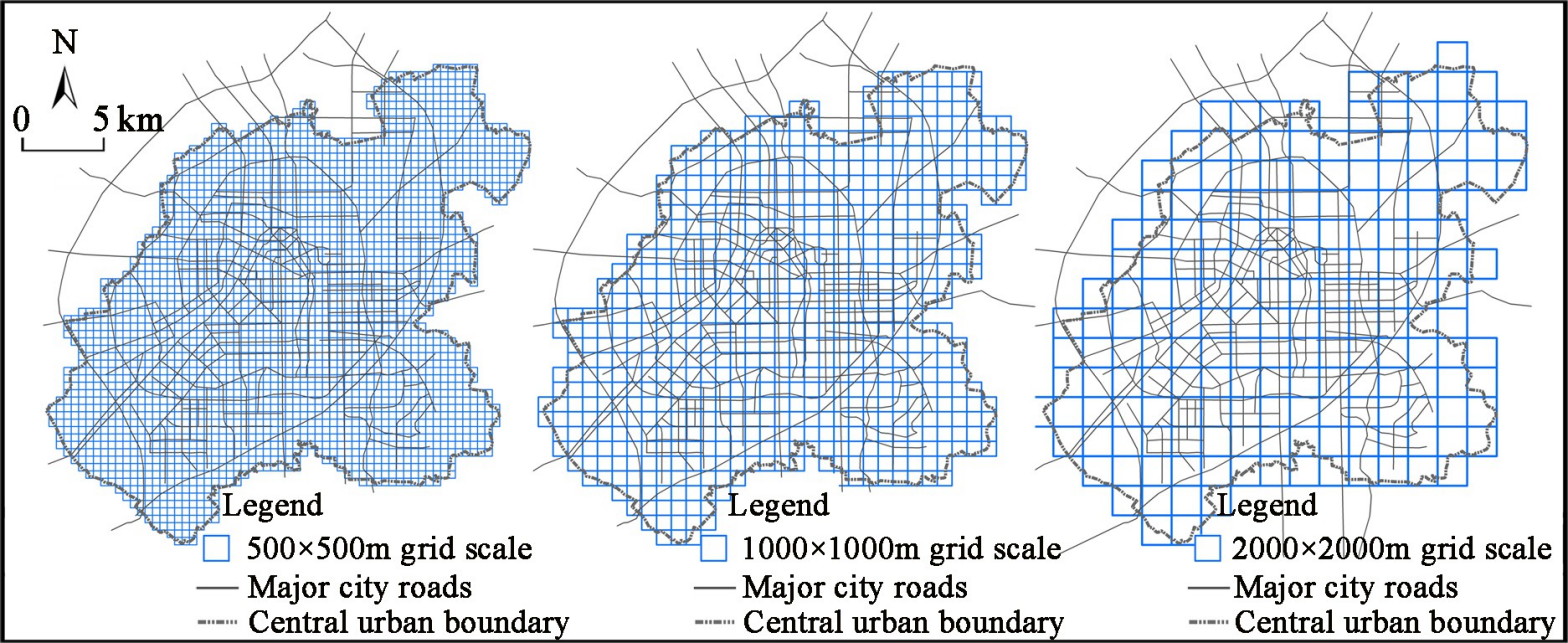
The central city area of Changchun City with the different scales of grid division.

Changchun, located in Jilin Province, China, is the capital city of the Jilin Province, and it is a sub-provincial metropolis, with a population of 4,866,500 in its urban area (excluding the Jiutai and the Shuangyang Districts) by the end of 2020, according to the Seventh National Population Census bulletin published by the Changchun Bureau of Statistics. Based on the long-term tracking research of our research group, we found that the development of urban landform in Changchun City shows a typical pattern of expanding circle, and the urban land structure and function problems encountered in its development process are typical of large cities in China. As the research scope of this paper, we have adopted the definition of the Changchun’s central urban area determined by the Changchun Urban Master Plan (Revised Version 2017), with an area of about 612 km2 (Fig 1). To analyze the coupling differences between the urban land structure and its function at different scales, a multi-scale fishnet division of the central urban area has been done. However, what has been found was that the scale of the fishnet was too large to reflect the basic characteristics of the urban land structure, while the measurement results were susceptible to the chance factors or even data errors in the cases where the scale of the fishnet was too small. After the repeated tests, three grid scales of 500×500 m, 1000×1000 m, and 2000×2000 m were selected to be used in the study, which can not only accurately portray the characteristics of the urban land structure and the coupling and the coordination relationships with its function, but which can also reflect the differences in the coupling relationship on different scales.

### Data sources

In this study, land density (*N*) and land mixed index (*M*) represent the land structure, while urban population distribution (*P*), the Baidu thermal distribution (*R*), the public service facility distribution (*A*), the business service facility distribution (*B*), and the public transportation station (*T*) represent the urban functions. The land use data is made from the land use status map of the Changchun city’s general planning, which is verified and supplemented by the remote sensing image comparison and field research, with the goal to form the basic land use data of the major urban functions in 2021. The population data comes from the 2020 census data. The Baidu thermal map as a data source can objectively reflect urban vitality and at the same time is easily assessable. Researchers and city managers can use it easily in order to grasp the real dynamic changes of urban vitality. Accordingly, it has been widely used in urban vitality research in recent years, and this study also used the Baidu thermal maps to reflect the level of urban functional development. Baidu thermal data is the intercepted data of the Baidu electronic map made at 18:00 h on May 19, 2022. After a rough comparison of multiple time periods, the overall average thermal value at 18:00 h on the Baidu electronic thermal map was higher than other time periods, with a wider data coverage and richer data volume. Public service facilities date are POI data from primary and secondary schools and hospitals in Changchun City in 2021. Business service facilities date are POI data of hotels (above 3-star) and four major state-owned banks (Industrial and Commercial Bank of China, Agricultural Bank of China, Bank of China, and China Construction Bank) located in Changchun in 2021. All public transportation stations date are obtained from the searchable public transportation stations of Baidu electronic map and Tencent electronic map in December 2021 (Table 1). The reason why both Baidu electronic map and Tencent electronic map were used in this study is because they can complement each other, i.e., the way different electronic maps present data is different, so the thermal map is unique to the Baidu electronic map, while the Tencent electronic map is relatively complete for various categories of service facilities. Thus, the acquisition of data for various categories of service facility networks based on both types of electronic maps can enhance the accuracy of the data used.

**Table 1:** Urban land structure, urban function measurement index selection, and data sources.

Data Category	Measurement index selection	Data source description
**Urban land structure** **(*U***_***1***_ **)**	Land density (*N*)	The land use status map, combined with the remote sensing images and field research to verify and supplement. Extract and vectorize 5 types of land: residential land, industrial land (industrial land and logistics land), commercial land, public service facility land (public service land and education and research land), green land and water.
	Land mixed index (*M*)	
**Urban function** **(*U***_***2***_ **)**	Urban population (*P*)	Census data.
	Baidu thermal map (*R*)	Baidu thermal map, spatially corrected to extract thermal property values.
	Public service facility (*A*)	Primary and secondary schools and hospital POI data overlay. Refer to the data published on the websites of Changchun Education Bureau and Health Care Commission.
	Business service facility (*B*)	Hotel and bank POI data overlay. Baidu electronic map and Tencent e-map acquisition.
	Public transportation station (*T*)	Baidu electronic map and Tencent electronic map acquisition, POI data.

### Methods

#### Land use density and the mixed land use function index

Land density (*N*) refers to the ratio of the major urban functions’ land area to the grid area in each grid, reflecting the scale and the concentration of the major construction land, and, at the same time, illustrating the scale and distribution characteristics of the dominant land functions in different grids by combining the land use status data.

There is a close relationship between the degree of the land use types mixing and the urban functions. The Mixed Index (*M*) can be used as a measure of the degree of mixing of different land types, and it can also reflect the degree of integration between different types of land more accurately, where the latter could be seen as an important indicator when determining the spatial pattern characteristics of different land types [32,34].

This study will combine land use density (*N*) and the mixed land use function index (*M*) to portray the spatial structure characteristics of the urban land use at different scales.

#### Exploratory spatial data analysis

Spatial autocorrelation is a classical method used in the study of the spatial agglomeration characteristics of geographic elements. It is widely present in the current research of the geographical spatial element patterns. The Local Indicators of Spatial Association (LISA) have been employed within this study to analyze the local spatial agglomeration characteristics of land density and the mixed function index at different scale grids, as well as to visualize the spatial agglomeration characteristics geographically. The main LISA calculation formula that has been employed is as follows [49]:

$$I_{i}=\left(\frac{Z_{i}}{\sum_{i}Z_{i}^{2}}\right)\sum_{j}W_{ij}Z_{j}$$ (1)

In the equation, *Z*_*i*_ and *Z*_*j*_ denote the reachability of the *i* and *j* grid cells and the mean deviation of the analyzed figures, respectively. W_*ij*_ denotes the proximity relation of *i* and *j* grid cells.

#### Coupling coordination degree

The concept of coupling refers to the relationship between two or more systems that exist in a close cooperation and mutual influence. It is now widely used in the socio-economic research field. For the further measurement of the coordination between the urban land use structure and the urban functions, the coupling coordination degree is constructed. The calculation formula is as follows:

$$D=\sqrt{\left(\sqrt{\frac{U_{1}\times U_{2}}{{\left(U_{1}+U_{2}\right)}^{2}}}\right)}\times (\alpha U_{1}+\beta U_{2})T$$ (2)

In the equation above, *U*_1_ represents the land use structure index (made out of the land use density, *U*_*1N*_, and land use mixed index, *U*_*1M*_), and *U*_2_ represents the urban function index (made out of the urban population, *U*_*2P*_, the Baidu thermal index, *U*_*2R*_, the public service facilities, *U*_*2A*_, the business service facilities, *U*_*2B*_, and the public transportation stations, *U*_*2T*_). *α* and *β* represent the weights, both taking the value of 0.5, and *D* is the coupling coordination degree.

The intent of this study has been to calculate the coupling coordination degree between the land density, *U*_*1N*_, the land use mixed index, *U*_*1M*_, the urban population, *U*_*2P*_ (*NPD*, *MPD*), the Baidu thermal index, *U*_*2R*_ (*NRD*, *MRD*), the public service facilities, *U*_*2A*_ *(NAD*, *MAD)*, the business service facilities, *U*_*2B*_ *(NBD*, *MBD)*, and the public transportation stations, *U*_*2T*_ *(NTD*, *MTD)*, respectively, in the grid at different scales, and all coupling coordination degrees have values in the range from 0 to 1. The calculation results are visualized for the further analysis of the coupling coordination relationship between the urban land use structure and its function. This research has constructed the abovementioned 10 indices, which are helpful for the quantitative analysis of the coupling relationship between the urban land use spatial structure and urban functions and are also an important methodological contribution of this research.

## Results

### Major functional space land use pattern characteristics

#### Land density pattern characteristics

**Fig 2:**
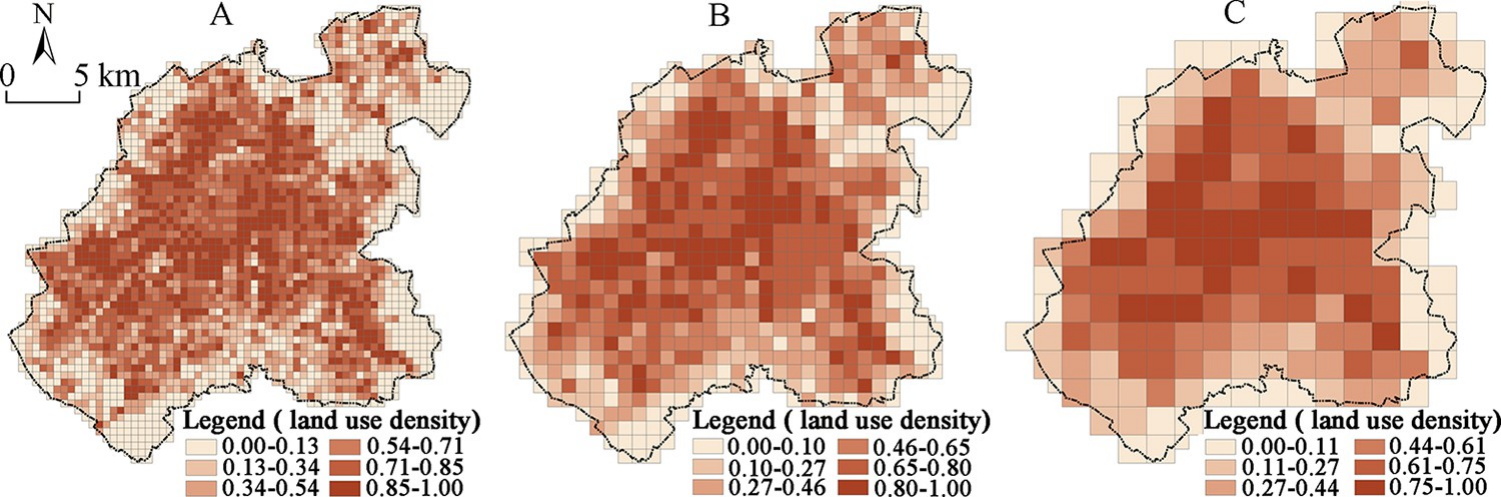
Density distribution of the construction land for the main functional spaces in the central urban area of Changchun at different scales: (A) 500 m scale; (B) 1000 m scale; (C) 2000 m scale.

From the density of the urban construction land in the grid of Changchun central city at the scales of 500×500 m, 1000×1000 m, and 2000×2000 m (Fig 2), the overall density of the urban construction land exhibits the spatial pattern characteristics of high density in the center and of low density in the periphery. At the same time, the high-density agglomeration area exhibits the distribution characteristics of “one core and many pieces”, i.e., the overall density of the land in the core area is high, except for the periphery of the Southeastern Jingyue District, the Southwestern Automobile Economic and Technological Development Zone, and the Northeastern Beihu District which also have a higher density of agglomeration area.

**Fig 3:**
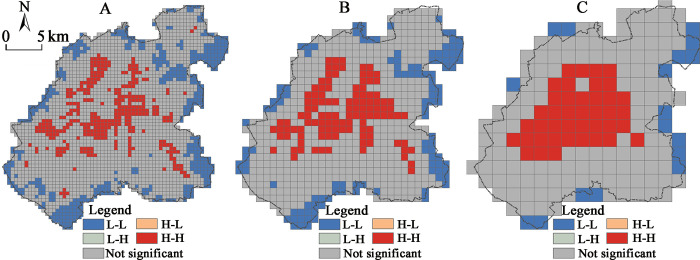
LISA distribution map of the main functional space construction land density in the central urban area of Changchun at different scales (p<0.1): (A) 500 m scale; (B) 1000 m scale; (C) 2000 m scale.

The spatial scale dependence of the density distribution of the major construction land in the city was found to be significant, and the spatial agglomeration characteristics to vary significantly at different scales, thus showing the basic characteristics of the significant agglomeration at large scales, and relative scattering at small scales. Among them, the 2000×2000 m scale grid has shown the most significant spatial agglomeration of the construction land density distribution (Fig 2C), and the density of the major construction land decreases from the center to the periphery. Moreover, the corresponding LISA distribution map has verified this spatial pattern more intuitively (Fig 3C).

The “high-high” significant aggregation area has been formed within the “three rings”, and the “low-low” significant agglomeration area appears to go along the two sides of the central city boundary at the periphery. High population and economy agglomeration in the core area is common in large cities in China and it promotes intensive use of land, resulting in a significantly higher land density in the core area than in the periphery. Although, at smaller scales, the density of the construction land has also exhibited the basic feature of the core area being higher than the periphery, the LISA analysis has shown that the areas with the significant spatial agglomeration were not concentrated in the core area. Furthermore, the LISA of the land density in the areas with dense transportation facilities, located along the railroads and the urban expressways has not been found as significant (Fig 3A and 3B). The reason behind this could be that the density of the urban land at smaller scales has been significantly influenced by the area occupied by the transportation and public service facilities. For example, the spatial agglomeration in the area around Changchun station is not significant at 1000×1000 m and 500×500 m scales, in contrast to the 2000×2000 m grid scale which seems to mask this spatial distribution feature.

In addition, the regional LISA “high-high” agglomeration seems to have occurred in the peripheral areas of the Jingyue District, the Automobile Economic and Technological Development Zone, and the Beihu District at the 500×500 m scale, reflecting the higher urban land density in the key development areas of the peripheral areas of the city, at a smaller spatial scale. From the perspective of small-scale land density, this could be seen as another indication of the central city exhibiting a certain polycentric spatial pattern.

#### Pattern characteristics in the mixed land use function index

**Fig 4:**
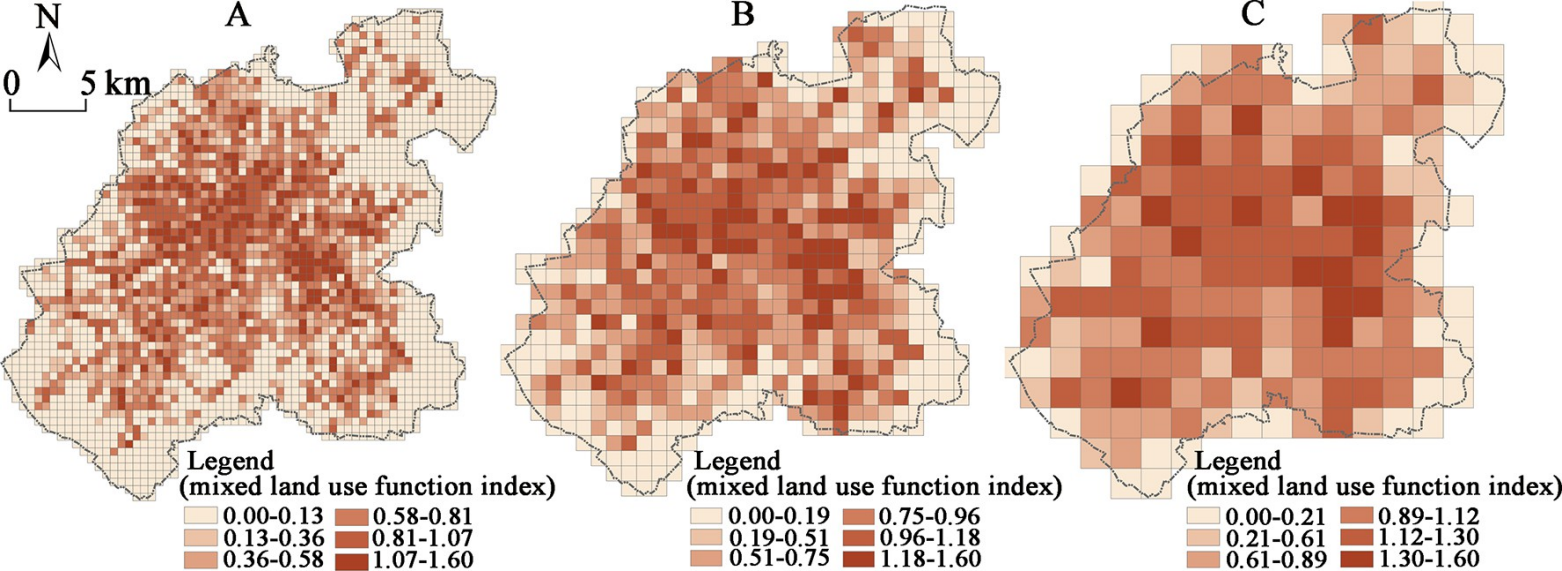
The distribution of the mixed land use function index of the main functional space in the central urban area of Changchun at different scales: (A) 500 m scale; (B) 1000 m scale; (C) 2000 m scale.

**Fig 5:**
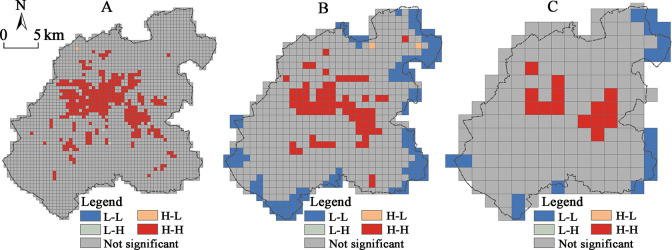
The distribution map of LISA of the mixed land use function index of the main functional space in the central urban area of Changchun at different scales (*p*<0.1): (A) 500 m scale; (B) 1000 m scale; (C) 2000 m scale.

The spatial distribution of the mixed land use function index has been detected as relatively scattered, as opposed to the distinctively different spatial pattern. The high value grid has not shown any significant urban core agglomeration characteristics, and the “mosaic” characteristics of the high value grid have been prominent, while the edge areas along the central city boundaries have had a continuous low value distribution (Fig 4). The areas with the high values of the mixed land use function index at the three scales have displayed a certain coherence: the core area of the “People’s Street–Liberty Road–Gongnong Road–Puyang Street (Youth Road)–Taipei Street” has been found to be a high-value aggregation area, within which both sides of the Chongqing Road Commercial Street haven’t been found as significant. It can be speculated that the main reason is the concentration of the service facilities leading to a single land use structure, i.e., the fact that the eastern part relies on the Economic and Technological Development Zone, roughly encircled by the “Yatai Street–Satellite Road–Jilin Road” area, which is a high-value agglomeration area. The functional mixed indices of the two areas mentioned above have been shown to have a more significant “high-high” agglomeration feature in the 500×500 m, 1000×1000 m, and 2000×2000 m scales of the grid LISA analysis (Fig 5).

Overall, the mixed land use function indices in the commercial areas with dense service facilities and the peripheral areas with more concentrated industrial and residential land use seem to be relatively low. The LISA analysis of the mixed land use function indices in the grids at the scale of 1000×1000 m and 2000×2000 m has both shown the presence of the “low-low” agglomeration (Fig 5B and 5C), indicating that the mixed function index of the land structure in the urban periphery has exhibited a sudden decrease. Combined with the analysis of the remote sensing images and the field research, the main reason for this pattern change could be found in the land structure of the urban periphery being more homogeneous, and the proportion of the industrial and the residential land being often higher, thus consequently affecting the degree of the mixed land function. The main reason for the absence of the significant LISA “low-low” agglomeration in the urban fringe area at the 500×500 m scale could be the overall land use structure of the urban fringe area being homogeneous at small scales, alongside the obvious randomness of the mixed land use function index distribution.

### Main functional space land use pattern and urban function coupling coordination relationship

#### Pattern differences in coupling coordination relationships

**Fig 6:**
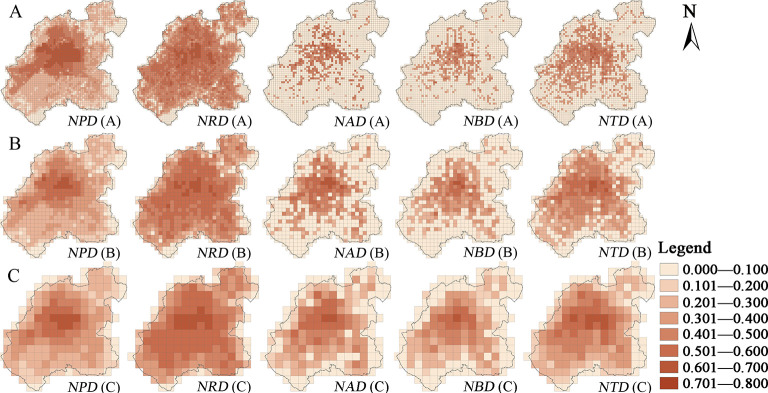
The distribution map of the coupling coordination degree of the land use density and the main urban functional elements in the central urban area of Changchun at different scales: (A) 500 m scale; (B) 1000 m scale; (C) 2000 m scale. Note: *N* is the land use density, *P* is the urban population, *R* is the Baidu thermal, *A* is a public service facility, *B* is a business service facility, *T* is a public transportation station, *D* is the coupling coordination degree. *NPD* represents the coupling coordination degree between the land use density and urban population, and so on.

**Fig 7:**
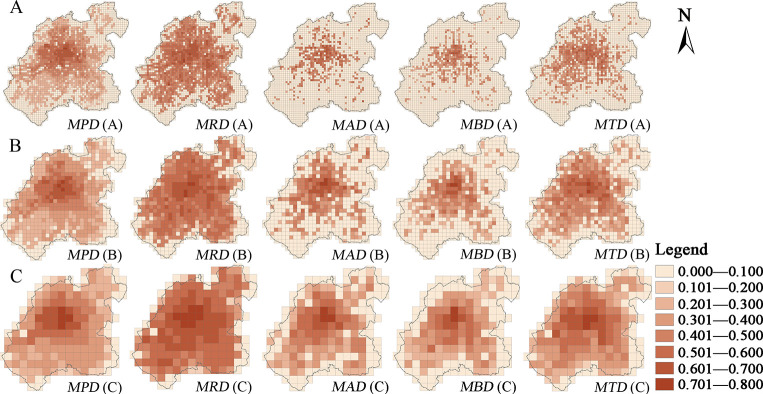
The distribution map of the coupling coordination degree between the mixed land use function index and the main urban functional elements in the central urban area of Changchun at different scales: (A) 500 m scale; (B) 1000 m scale; (C) 2000 m scale. Note: *MPD* represents the coupling coordination degree between the mixed land use function index and the urban population, and so on.

When it comes to the land use pattern of the main functional space and the spatial pattern of the coupling coordination of the urban functional elements, the overall found trend was a gradual decrease from the urban core to the periphery (Figs 6 and 7).

The core of the urban area has exhibited a high density of the construction land and the relatively diversified types of land functions, which support a dense population and the service facilities, etc. The corresponding urban Baidu heat value has also been found to be generally high, indicating that the spatial structures of the compact and the diversified land use are a reflection of the more ideal urban functions. From the urban center to the periphery, the density of residential, the service facilities, and even the industrial land has been shown to gradually decrease, coupled with a large-scale emergence of the residential areas, industrial parks, and the science and education parks under the guidance of the urban functional zoning planning ideas. What is more, the land function mixed index, in turn, has been shown to decrease, although the land density of some peripheral urban areas has seemed to reach a high level, such as the Southern New District, the Southwestern Automobile Economic and Technological Development Zone (mainly concentrated in the FAW factory area), and the Changchun New District (mainly refers to the northeastern part of Changchun), the mixed land use function index has been found to be relatively low, as well as the coupling coordination between the land density of the urban main functional space, the mixed land use function index, and the functional elements. These findings could indicate that the degree of the ideal urban functions is closely related to the urban land use structure, meaning that an intensive and a diversified land use structure is more likely to support an ideal functional urban area, while a coarse and a single land use spatial structure seems to be often inadequate for the population gathering. The relatively high proportion of the service-facility and the residential land in the urban core could be pointing out that the land structure type with its service and residential functions as the dominant ones is, in fact, a reflection of relatively ideal urban functions. On the other hand, the type of land structure whose dominant functions are the industrial land, green space, and water would then be a reflection of the relatively low urban functions. Over the past 30 years, various new cities and districts have been established in China’s big cities. The reason why many of them are called "empty cities" and "ghost cities" is that many new areas in China were initially set up with excessive consideration of industrial or residential functions, and the land use structure in urban planning is homogeneous, leading to underdevelopment of urban functions and consequently affected the population and economic agglomeration.

In terms of the spatial pattern, the local aggregation changes of the land density and the mixed function index have not shown a significant impact on the coupling coordination of the urban land structure and function. Although a local low value aggregation of the land density has been found along the railroad station and the railroad line in the old district, together with a local high aggregation value of the mixed land use function index in the Economic and Technological Development Zone, the coupling coordination between the various elements in these two has not shown any significant high or low value aggregation characteristics. This could be an indication that the local changes of the land density and the mixed land use function index have relatively insignificant spatial impact on the urban function.

#### Category differences of coupling coordination relationship

The coupling coordination degrees (*NPD*, *MPD*) of the urban population distribution, the land use density and the mixed function index have exhibited the typical high value agglomeration characteristics of the core area, which could be mainly attributed to the high land use density and the mixed function index in the old city, which is also the most densely populated area with the People’s Square as its core (Figs 6 and 7). Although the land density and the mixed function indices have been found to be higher in the area within the “second ring” than the ones within the “fourth ring” from the periphery of the core area, the coupling coordination degree has also been found as significantly lower. The main cause could be the significant decrease of the population density. Although the coupling coordination degree (*NRD*, *MRD*) between the Baidu thermal value, the land use density, and the mixed function index have also exhibited an overall trend of the gradual “core-periphery” decrease, the coupling coordination degree values have been found to be generally higher than other elements, which could be mainly due to the relatively uniform spatial layout of the Baidu thermal map, as well as the distribution of the values which possess certain smoothness characteristics. The spatial coupling coordination degree (*NAD*, *MAD*) of the public service facilities, the land density, and the mixed function index, along with the spatial coupling coordination degree (*NBD*, *MBD*) of the business service facilities, and their land density and mixed function index have been shown as relatively consistent, with the overall coupling coordination degree being low. However, the location of the areas with the coupling coordination degree higher than 1 are the old district, which is within the “third ring” and the core functional area of Jingyue. This could be explained by the spatial distribution of the public and business service facilities having a significant agglomeration feature, and the layout of various service facility outlets failing to keep up with the expansion of the construction land, thus resulting in the spatial imbalance of the land structure and the urban service functions. The overall spatial coupling coordination degree (*NTD*, *MTD*) of the public transport stations and their land density and mixed function index has been found to be at a medium level. Compared with the public and business service facilities outlets, the areas with a high coupling coordination degree have been shown to be more widely distributed, mainly due to the public transport stations having been mostly laid out in the periphery of the urban area, under the guidance of government planning, before the public and business service facilities were too. In contrast, the coupling coordination degree of the public transport stations and the land structure in the periphery of the central city has been found to still be low, compared with the population and the Baidu thermal, stipulating that there could still be a lack of the public transport station settings in the periphery of the city.

#### Scale differences of the coupling coordination relationship

The coupling between the land use structure and its function in the core area of the city has been shown as high and not significantly influenced by the local land use structure. The coupling coordination degree of each type has also shown a more significant trend of the “core-periphery” distribution from high to low at different scales (Figs 6 and 7). Some differences have been uncovered in the coupling coordination degrees of the land density, the mixed land use function index, and the urban function at different scales. The large scale (2000×2000 m) has reflected the overall characteristics of the coupling coordination from the core to the periphery, which reaffirms the correspondence between diverse land use structure and ideal urban functions in the core area, while various new areas on the periphery of the city reduced the level of land use diversity due to excessive emphasis on functional zoning, which corresponds to the relative lack of ideal urban functions. The small scale (500×500 m) seems to have been heavily influenced by the occasional factors. The distribution of the high value grid of the coupling coordination also seems to be more scattered, even in the urban core area within the “second ring”. Moreover, the coupling coordination degree has also exhibited the low value “mosaic” distribution characteristic, which could be due to the large area of a single land type. Finally, the peripheral Jingyue District, the Southern New District, and the northeastern part of Changchun have also been shown to have a certain distribution of the high values of the coupling coordination degree, which could be an obvious sign of the urban land structure and its functions having a high coupling coordination degree in the small-scale peripheral areas and polycentric characteristics of the spatial pattern of the urban functions in the small-scale view.

## Discussion

Based on the results of our research combined with the implications of the successful cases of the existing large cities’ spatial structure optimization, we could draw the following three suggestions for the transformation and a high-quality development of the large cities’ functional space.

Firstly, more attention should be paid to the notion of building both an intensive and a mixed urban functional spatial structure. More specifically, what should be taken into consideration, within the urban spatial planning, are the diverse needs of the residents, just as the organic integration of urban functions which should receive more attention. Furthermore, the coordination of the human-land relations should be improved, while the “pendulum” commuter traffic and the social “cracks” caused by excessive zoning should be avoided.

Secondly, it should be noted that the spatial layout of the service facilities is related to people’s well-being, and the lag in the planning and construction of those service facilities has become an important reason for the poor coupling coordination between the land space structure and the urban functions. The rapid expansion of the urban land use and the high core concentration of various service facilities have also been shown to form a stark contrast, which has consequently led to the unbalanced service space in the big cities, and the inadequate access to the service facilities for residents. Therefore, the future urban planning and construction should be devoted to the coordinated promotion of the urban land use space and the service space, having in mind the coordinated layout of the service facilities needed for achieving a balanced and an adequate development of the big cities’ functional space.

Thirdly, even though the polycentric urban spatial structure is considered to be an effective way to disperse the urban functions, in practice, the construction process of a polycentric urban spatial structure in many large cities in China has been slow. The findings of this study point out that the polycentric urban spatial structure of Changchun has so far only been reflected at the small scale, opposite the large scale, which has not shown the formation of the polycentric spatial structure. What is more, the local change of the land use structure has not formed a significant impact on the urban function spatial distribution, even though the polycentric urban spatial structure should not only be reflected on the land use level, but also on the functional level, through the “people-land” interaction.

The shortcomings of this research mainly include the inability to conduct a longitudinal study of the dynamic coupling relationship between the urban land use structure and its function due to data unavailability and the space limitation, combined with the incapacity to establish a more accurate statistical model of the urban “structure-function” association. Therefore, we encourage future studies to further explore this association.

## Conclusions

The results of the study show that both the land use density and the mixed land use index have exhibited the basic characteristics including the core area being higher than the periphery as a whole, the land use density being significantly agglomerated at the large scales and relatively scattered at small scales, the scale differences in the distribution of the mixed land use index not being obvious, and the concentration areas of the LISA “high-high” values being present in the core areas at multiple scales.

This study reflects the degree of mixed urban land use through land use density and land use diversity index, and the research confirms a significant positive correlation between the diversity of urban land use structure and function. When it comes to the pattern differences of the coupling coordination degree of the urban land use structure index and the function index, its spatial pattern as a whole has shown a trend of a gradual decrease, from the core to the periphery, indicating the possibility of an intensive and a diversified land use structure, thus supporting the model functional urban area being high. Furthermore, the diversity of urban land use structure contributes to the improvement of urban functions.

When it comes to the category differences of the coupling, the coupling coordination of the population distribution, the Baidu thermal distribution, and the land structure is relatively high, which indicates that the diversity of urban land structure contributes to the agglomeration of population and thus presents a higher level of urban vitality. This is followed by a slightly lower value of the public transportation stations, while the public and business service facilities proved to be relatively low, that is, the spatial match between various service facilities and the diversity of land structure is low. This can be taken as a reflection of the relative lag in the construction of the service facilities, which could also be an important reason for the spatial imbalance of the large cities, and the improvement of various service facilities on the periphery of cities becomes an important way to improve the coupling of land structure and function in large cities. It should be noted that the overall coupling coordination degree between the Baidu thermal distribution and the land use structure has been shown as high with the high spatial continuity characteristics, thus reflecting the advantages of the Baidu thermal data in portraying the functional space of the large cities, illustrating its high application value.

Finally, when it comes to the scale differences of the coupling, the overall change of the coupling coordination degree from the core to the periphery has been exhibited more significantly at the large scales, while the polycentric characteristics of the urban function spatial pattern have become obvious at small scales. These results indicate that the local fine-tuning of urban land structure has no significant impact on urban function, and the overall diverse pattern of urban land use can support ideal urban functions, and the enhancement of urban vitality requires the rational layout of urban land use structure as a whole. Moreover, the coupling coordination degree of the urban land use spatial structure and its function has been found to be sensitive to the spatial scales, leading to the conclusion that the importance of the scale in the urban spatial research deserves more attention.

Optimization of urban functional space is a long-standing research topic in urban geography and urban planning, and this study is a continuation of existing research. Compared to existing literature on spatial patterns and functions of land use in large cities, this research is unique in that it classifies and compares urban land use structure with other major functional spatial structures, quantifies the coupling coordination relationship between urban land use structure and urban functions, and confirms the positive spatial correlation between the diversity of urban land use structure and other major urban functions. It also reveals the variability of urban population, urban vitality, and the coupling of different service facilities with the urban land use structure, which provides valuable references for future planning related to the strengthening of urban vitality.

## Supporting information

S1 Table 500m scale main data of each grid. (XLSX)

S2 Table 1000m scale main data of each grid. (XLSX)

S3 Table 2000m scale main data of each grid. (XLSX)
